# Characterizing properties of non-estrogenic substituted bisphenol analogs using high throughput microscopy and image analysis

**DOI:** 10.1371/journal.pone.0180141

**Published:** 2017-07-13

**Authors:** Adam T. Szafran, Fabio Stossi, Maureen G. Mancini, Cheryl L. Walker, Michael A. Mancini

**Affiliations:** 1 Molecular and Cellular Biology, Baylor College of Medicine, Houston, Texas, United States of America; 2 DeepBio, Inc., Houston, Texas, United States of America; University of Wisconsin Madison, UNITED STATES

## Abstract

Animal studies have linked the estrogenic properties of bisphenol A (BPA) to adverse effects on the endocrine system. Because of concerns for similar effects in humans, there is a desire to replace BPA in consumer products, and a search for BPA replacements that lack endocrine-disrupting bioactivity is ongoing. We used multiple cell-based models, including an established multi-parametric, high throughput microscopy-based platform that incorporates engineered HeLa cell lines with visible ERα- or ERβ-regulated transcription loci, to discriminate the estrogen-like and androgen-like properties of previously uncharacterized substituted bisphenol derivatives and hydroquinone. As expected, BPA induced 70–80% of the estrogen-like activity via ERα and ERβ compared to E2 in the HeLa prolactin array cell line. 2,2’ BPA, Bisguaiacol F, CHDM 4-hydroxybuyl acrylate, hydroquinone, and TM modified variants of BPF showed very limited estrogen-like or androgen-like activity (< 10% of that observed with the control compounds). Interestingly, TM-BFP and CHDM 4-hydroxybuyl acrylate, but not their derivatives, demonstrated evidence of anti-estrogenic and anti-androgenic activity. Our findings indicate that Bisguaiacol F, TM-BFP-ER and TM-BPF-DGE demonstrate low potential for affecting estrogenic or androgenic endocrine activity. This suggest that the tested compounds could be suitable commercially viable alternatives to BPA.

## Introduction

The impact of man-made chemicals on human health and the environment is a continuous global concern. Between the thousands of compounds synthesized, chemicals that impact the endocrine system took a central stage as they have the potential to affect whole live cycle of humans through central functions like reproduction and metabolism, while accumulating over time both in the human body and the environment. Probably the best studied endocrine disrupting chemicals (EDCs) are the ones with estrogenic or anti-estrogenic activity. Of these, Bisphenol A (BPA) has garnered attention due to its widespread detection in the human population and the environment, observed effects in laboratory animals, and plausible impact on human health. BPA is among the top 2% of organic chemicals produced commercially and is used in a vast variety of applications including many day-to-day products containing polycarbonate plastics and epoxies. When BPA is used to make polymers for lining of cans or bottles, it often leaches out into food or beverages, and is then ingested in microgram quantities [1–3]. Given its prevalence, it is of little surprise that biomonitoring studies detected BPA in most humans [4]. Based on its mode-of-action and results from rodent studies, BPA has been implicated in hormone-dependent cancers (*e*.*g*., breast and prostate), metabolic diseases (*e*.*g*., diabetes and obesity), developmental defects, and changes in fertility, neurological function, and behavior [5–7].

In light of health and environmental concerns about the safety of BPA, the demand for non-BPA-containing materials is high and the search for viable BPA alternatives has grown dramatically. Unfortunately, bisphenol alternatives such as bisphenol S (BPS) and bisphenol F (BPF) have scant health and epidemiological data; however, they may have a similar mode-of-action to BPA, including endocrine disruption. In fact, a recent systematic review of 25 *in vitro* and 7 *in vivo* studies concluded that BPS and BPF have activity and potency that is similar to BPA with estrogen-, anti-estrogen-, androgen-, and anti-androgen-like features [8].

Finding BPA replacements that can be used in commercial applications such as coating material for canned food packaging is challenging since bisphenol polymers have inimitable physical properties that are not available from non-bisphenol materials such as olefins, acrylics or polyesters [9]. Bisphenol-based epoxy polymers are able to maintain integrity for long periods of time across a spectrum of applications, from easy open cans to twist-off bottle closures. Previously, no equivalent to bisphenol polymer chemistry had been found that is equal to BPA for preventing can lining failure and subsequent food contamination, especially with acidic or fatty foods [10].

Multiple cell-based *in vitro* assays have been developed to measure estrogenic potential of chemicals, with the most common being estrogen-dependent cell proliferation and gene expression. The measurement of estrogenic activity based upon breast cancer cell proliferation (E-screen assay) and luciferase-based reporter gene activation that is under the control of estrogen-responsive enhancer elements (BH1Luc4E2 cell line) has proven to be robust and highly sensitive, resulting in their consideration by multiple agencies (EPA, ICCVAM/NICEATM, and OECD) for national and international estrogenic activity standards [1,11]. However, individually, these assays provide little mechanistic insight to differentiate between similar chemical compounds with potential estrogenic activity. Recently, efforts from NIEHS and EPA has led to the development of mathematical models that incorporate data from 18 different ToxCast *in vitro* assays that measure ER binding, dimerization, DNA binding, transcriptional activation and cell growth [12]. Importantly, this model was shown to be able to accurately predict the results of the classical estrogenic *in vivo* mouse uterotrophic assay, thus leading towards a demonstration that *in vitro* high throughput (HT)/high content analysis (HCA) platforms could be used as a substitute for very expensive and slow animal models.

**Fig 1:**
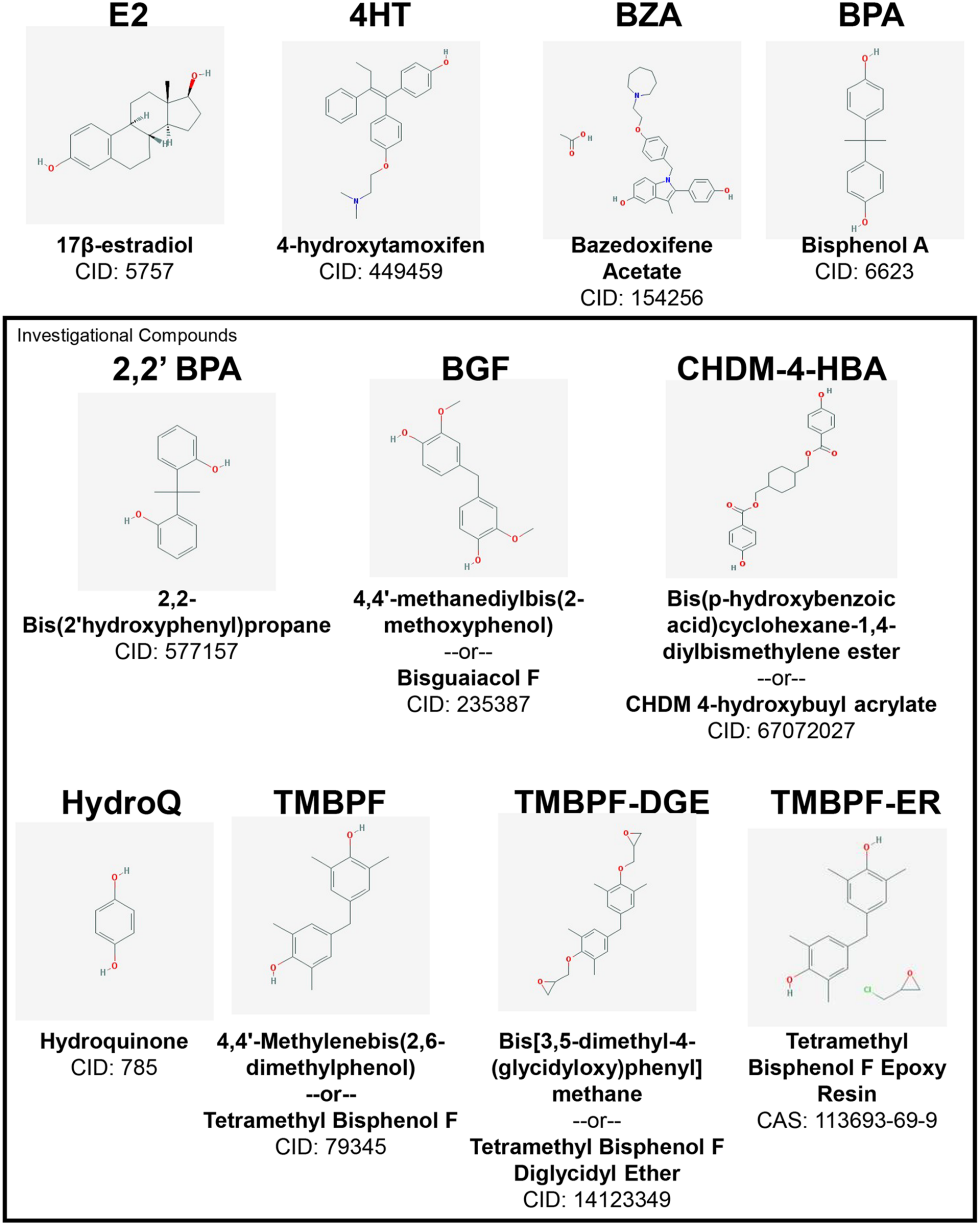
Chemical structures and identification numbers for compounds analyzed. Each compound is identified by an abbreviation, full name, and a PubChem compound identification (or CAS) number.

Compounds such as BPA that mimic or antagonize the *in vivo* or *in vitro* activity of the hormone 17β-estradiol (E2) are typically described as having either estrogenic (agonistic) or anti-estrogenic (antagonistic) activity. This is largely mediated by interactions with one or both of the estrogen receptor subtypes, estrogen receptor-alpha (ERα) or estrogen receptor-beta (ERβ), which are ligand activated nuclear transcription factors. We previously described the generation of a human cell line containing a stable, microscopically-visible, multi-copy integration of the ER-responsive prolactin promoter-enhancer unit (PRL-HeLa; Supplemental Materials, S1 Fig). This cell line, following expression of GFP-ERα, allows for direct and simultaneous visualization and quantitation of ER DNA binding, recruitment of coregulators, epigenetic mark alterations, chromatin remodeling, and transcriptional regulation in response to ER ligands [13–15]. The PRL-HeLa cell line was further-adapted to high throughput microscopy-based screening by the generation of stable variants expressing either ERα or ERβ translationally-fused with green fluorescent protein (GFP-ERα:PRL-HeLa and GFP-ERβ:PRL-HeLa). When combined with our custom, automated image analysis platform [16], these cell lines were used to discriminate and classify the mechanistic effects of E2, ER antagonists, BPA and 18 closely related BPA analogs on ERα and ERβ, a dataset that has also been included in the EPA mathematical models [12,17,18]. In the present study, we use these ERα- and ERβ-expressing PRL-HeLa, the widely utilized ER-positive MCF-7 breast cancer, and the AR-positive LNCaP prostate cancer cell lines in high content/high throughput assays to characterize and classify seven, less well studied, potential BPA substitutes identified by Valspar during an internal yeast-based *in vitro* assay (data not published). The molecules that we analyzed include: 2,2’ BPA, bisguaiacol F (BGF), CHDM 4-hydroxybuyl acrylate (CHDM-4-HBA), hydroquinone (HydroQ), tetramethyl bisphenol F (TMBPF), tetramethyl bisphenol F diglycidyl ether (TMBPF-DGE), and TMBFP-ER (Fig 1).

## Material and methods

### Chemicals

Structures, abbreviations, and PubChem ID numbers for all compounds used in this study are shown in Fig 1. 17β-estradiol (E2, CAS 50-28-2), 4-hydroxytamoxifen (4HT, CAS 65213-48-1), bazedoxifene acetate (BZA, CAS 198481-33-3), and 2,2-bis(4-hydroxy-3,5-dimethylphenyl)propane (TM-BPA, CAS 227-033-5) were obtained from Sigma. Certified bisphenol A (BPA, CAS 80-05-7) was obtained from Battelle. 2,2’ BPA (CAS 7559-72-0) was obtained from Toronto Research Chemical. BGF (CASN 3888-22-0), CHDM-4-HBA (CAS N/A, PubChem CID 67072027), HydroQ (CAS 123-31-9), TMBPF (CAS 5384-21-4), TMBPF-DGE (CAS 93705-66-9), and TMBPF-ER (CAS 113693-69-9) were obtained from Valspar. These compounds are known reference compounds or investigational compounds previously identified as non-estrogenic using a yeast-based screening method performed internally by Valspar (*unpublished data*). All chemicals were solubilized in ethanol (E2, BPA, TM-BPA, 2,2’ BPA, BGF, TMBPF, TMBPF-ER, TMBPF-DGE) or DMSO (BZA, CHDM-4-HBA), aliquoted, and stored at -20°C until use.

### Cell culture and treatments

The GFP-ERα:PRL-HeLa and GFP-ERβ:PRL-HeLa cell lines were grown in phenol red-free Dulbecco’s modified Eagle’s medium (DMEM) containing 10% fetal bovine serum (FBS; Gemini Bioproducts), L-glutamine, sodium pyruvate, 0.8 μg/ml blasticidin, 200 μg/ml hygromycin, and 10 nM 4HT (GFP-ERα:PRL-HeLa, Sigma) or raloxifene (GFP-ERβ:PRL-HeLa, Sigma). MCF-7 ERE-MAR cells were grown in phenol red-free DMEM containing 10% FBS, L-glutamine, and sodium pyruvate. 2PB-mCherry-NLS:LnCaP cells were grown in DMEM/F12 containing 10% FBS, L-glutamine, sodium pyruvate, 700 ng/ml puromycin, and 1 nM casodex. All cells were grown in treatment media (phenol red-free DMEM or DMEM/F12 containing 5% stripped/dialyzed FBS, L-glutamine, and sodium pyruvate and no drugs) for at least 48 hr prior to use.

For PRL-Hela or 2PB-mCherry-NLS:LnCaP experiments, cells were robotically seeded using a Titertek dispenser in 384-well optical Cyclo-Olefin-Polymer (COP) bottom plates (384-IQ, Aurora Biotechnologies) that have been empirically-shown to be free of estrogenic activity. Cells were allowed to adhere overnight prior to treatment with compounds alone or in combination with 10 nM E2 or DHT at concentrations ranging from 50 pM to 5 μM for either 1 hr or 24 hr.

For ERE-luciferase experiments, MCF-7 ERE-MAR cells (containing a stably integrated ERE-driven luciferase reporter gene) were manually plated into 24 well plates. Cells were allowed to adhere overnight prior to treatment with compounds alone at 2 μM and 5 μM. For combination experiments, cells were treated in the presence of 10 nM E2 for 24 hrs.

### Immunofluorescence

Immunofluorescence was performed as previously described [17,19]. Cells were fixed in 4% EM-grade formaldehyde in PEM buffer (80 mM potassium PIPES [pH 6.8], 5 mM EGTA, and 2 mM MgCl_2_) and quenched with 0.1 M ammonium chloride for 10 min. For samples in which no antibodies are used, cell membranes were permeabilized using 0.5% Triton X-100 for 10 min and DNA stained using DAPI (1 μg/ml) for 10 min. For samples with antibody labeling, permeabilization was with 0.5% Triton X-100 for 30 min. Cells were incubated at room temperature in blotto (5% milk in Tris-buffered saline/Tween 20) for 30 min, and then specific primary antibodies were added overnight at 4°C prior to 1 hr of secondary antibody (Alexa 647 conjugates; Molecular Probes) and DAPI staining (1 μg/ml for 10 min). The primary antibodies used were: mouse anti-Ser5-phospho RNA polymerase II (Abcam, ab5401) and mouse anti-SRC-3 (BD Transduction Labs No. 611105).

### High throughput microscopy and image analysis

Image data sets were collected using the IC-200 image cytometer (Vala Sciences, San Diego, CA) utilizing dual-step high-speed (50–100 ms) reflection- and image-based autofocusing, either a 40X/0.95 Nikon S-fluor or a 20X/0.75 Plan Apo objective, and a sCMOS 5.5-megapixel camera and LED illumination. Z-stacks were collected at 1 μm (20X) or 0.5 μm (40x) intervals and maximum-projected using the instrument-supplied Mexican Hat 13 x 13 synthetic focus algorithm. Cell, nucleus, array segmentation and signal quantification was performed using the myImageAnalysis web application powered by Pipeline Pilot software (Biovia) as previously described [16]. Aggregated cells, mitotic cells, and apoptotic cells were removed using filters based on nuclear size, nuclear shape, and nuclear intensity.

### MCF-7 ERE-MAR luciferase assay

MCF-7 ERE-MAR (also known as MCF-7-XVA2-Luc) cells, a kind gift from Dr. Steffi Oesterreich (University of Pittsburgh), are a human breast cancer cell line that contains a stable integration of the firefly luciferase gene under control of a promoter containing the estrogen response element (ERE) and matrix attachment region (MAR) derived from the *Xenopus* vitellogenin A2 promoter and responds to estrogenic compounds with a 30-35-fold induction of firefly luciferase [20]. After 24 hr of compound treatment, culture medium was aspirated and plates were frozen at -20°C until further processing. Cells were lysed with 1% Triton X-100, 10% glycerol, 2 mM EGTA, and 1 mM DTT. Luciferase was then measured using an automated microplate luminometer with the Promega Luciferase Assay System. Results from 4 replicates were collected and normalized to controls wells treated with E2 only.

### Calculation of estrogenicity (%E2) and statistical analysis

The estrogenicity of compounds was calculated relative to the maximum response observed with 10 nM E2 treatment (%E2, a measure of response amplitude). Each compound at each concentration was measured in triplicate or quadruplicate. Basal activity of each assay was determined using “mock” control (MC) containing treatment media and vehicle controls (VC) containing 0.5% DMSO solvent in a minimum of eight samples on each assay plate. The MC was set to 0% resulting in a %E2 calculation (1):

$$\%E2=100\%\times \frac{Response_{Sample}-Response_{MC}}{Response_{E2}-Reponse_{MC}}$$ (1)

Typical values for VC were 0% ± 5% E2. Inclusion of a VC accounts for any extraneous residual estrogenicity that might exist in the media or derived from the materials used for sample preparation. A chemical is considered to be significantly different from VC controls if it produces a mean %E2 significantly greater (p < 0.05) than VC as determined by ANOVA and post-hoc pairwise comparisons made using the Tukey HSD method. A compound is considered to have inhibited the E2 induced response if, when added to 10nM E2, it produces a mean %E2 significantly less than E2 controls. As a general rule, we consider a compound to have significant estrogenic properties with a %E2 score greater than 10% when used alone or anti-estrogenic properties with a %E2 change greater than 15%. %DHT calculations were done in an identical manner using 10 nM DHT at the control response.

Averaged results from a minimum of 4 independent experiments were graphed ± standard deviation (SD) using SigmaPlot software with EC_50_ values determined by fitting a 4-parameter sigmoidal curve. All data used to generate summary data can be found in a supplemental data file (S1 Data). Clustering analysis was performed using Cluster 3.0 and visualized with Java Treeview.

## Results

### Measure of estrogenic activity using ERα PRL-HeLa cells

**Fig 2:**
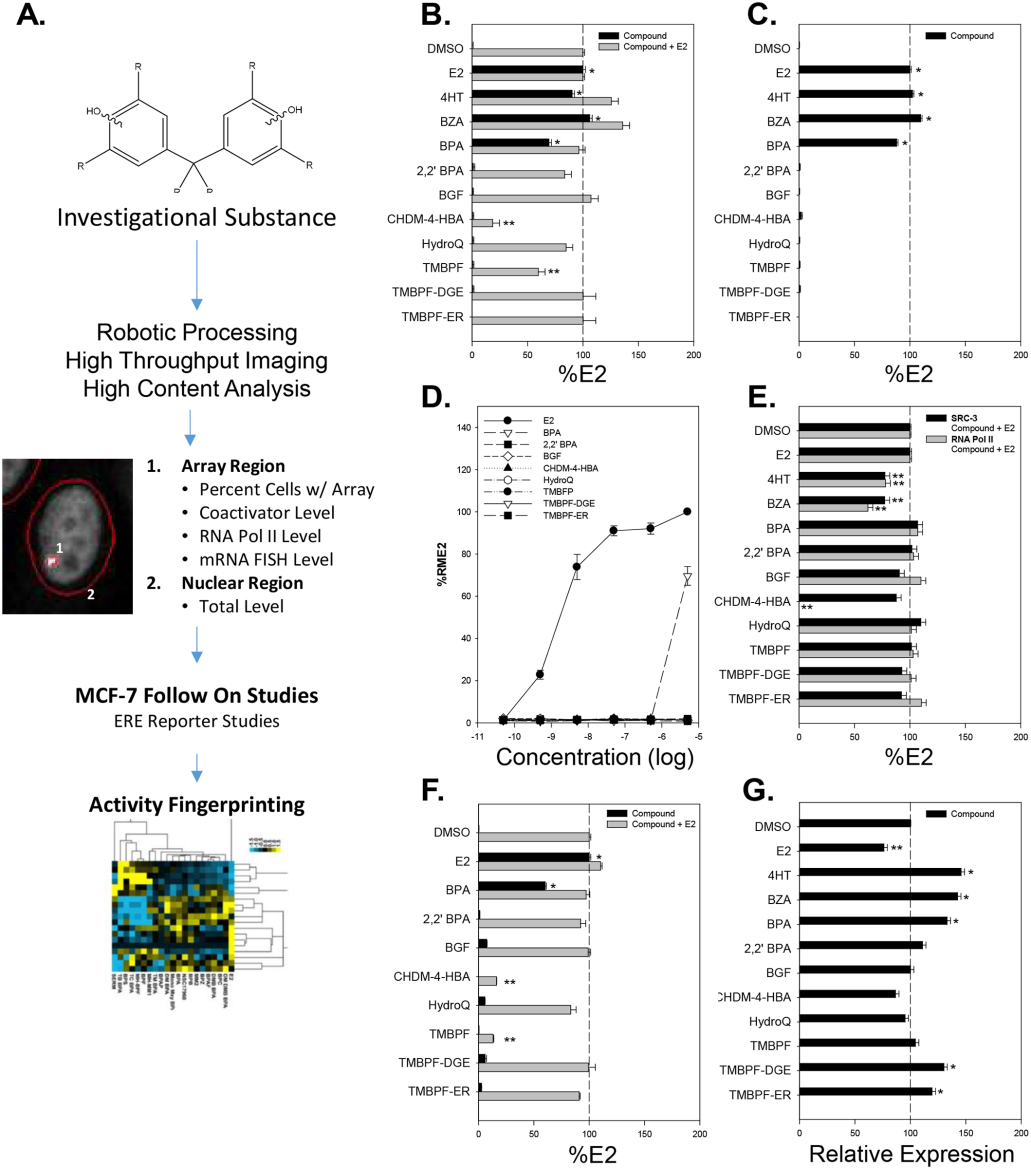
PRL-HeLa analysis of estrogenic and anti-estrogenic activity of investigational chemical compounds on ERα. (A) Experimental workflow for analysis of investigational molecules. Percentage of cells with an array in GFP-ERα:PRL-HeLa cells after 1 hour of treatment with maximal dose of compound alone or with 10 nM E2 after 1 hour (B) or 24 hours (C) of exposure. Antagonism experiments were done combining 10nM E2 with 5 μM of the compounds of interest. (D) Dose-response curves of percent of cells with arrays for E2, BPA and the seven investigational compounds. (E) Effects on SRC-3 coactivator and RNA polymerase II levels at visible arrays after 1 hour of treatment with 10 nM E2 in the presence of maximal dose of compound. (F) Effects on de novo mRNA production at the PRL array after 1 hour of treatment with compound alone or with 10 nM E2. (G) Effects on GFP-ERα expression level after 24 hours of treatment with maximal dose of compound. All data represent mean of a minimum of 4 experiments ± standard deviation. (*) or (**) indicates a response significantly (p < 0.05) above or below control treatment.

As described in the introduction, we previously established the utility of an engineered PRL-HeLa cell line, stably-expressing either GFP-ERα or GFP-ERβ, for high content analysis of ER-mediated transcriptional regulation and classification of endocrine disruptor chemicals (S1 Fig) [13,17]. Fig 2A describes the overall workflow of the high throughput assays and analysis routines that were used in the present analysis.

We initially assessed the ability of the potential endocrine disrupting compounds (Fig 1 for abbreviations used, CID #, and structures) to induce GFP-ERα recruitment to the multi-copy ERE in the PRL array, as compared with E2 10nM, which is always used as a positive (agonist) control. Also, in all experiments, the selective estrogen receptor modulators (SERMs) 4HT and BZA are used as antagonist controls. ER recruitment to the PRL array reflects a combination of ligand binding, receptor dimerization and DNA binding and was assessed after 1hr of treatment. Fig 2B shows the percent of cells with visible GFP-ERα targeting the ERE-rich promoter locus. Consistent with our previous work, treatment with ER SERMs such as 10 nM 4HT or BZA produced a DNA binding response equivalent to that of E2 alone (%E2, 90% and 106% respectively), consistent with the understanding that ER bound to SERMs bind DNA but recruit co-repressor proteins rather than co-activator proteins. Treatment with 5 μM bisphenol A (BPA) also induced significant DNA binding, although with lower efficacy as indicated by a %E2 of 69%. With 1 hr treatment at 5 μM, we were unable to observe any significant induction of GFP-ERα DNA binding (%E2 < 1%) by any of the seven investigational compounds (BGF, 2,2’ BPA, CHDM-4-HBA, HydroQ, TMBPF, TMBPF-DGE, or TMBFP-ER). Similar results were observed when treatment time was extended to 24 hr (Fig 2C) or concentrations ranging between 50 pM to 5 μM were tested (Fig 2D). Concentrations beyond 5 μM resulted in significant cell toxicity and were not analyzed.

As expected, treatment of cells with E2 and either ER SERM did not decrease the DNA binding as receptor bound to either the agonist or the SERM demonstrates high affinity for estrogen responsive promoter elements. Interestingly, two of the investigational compounds (CHDM-4-HBA and TMBPF) had significant effects when combined with E2 as they reduced the % of cells with visible arrays from 100% to ~60% (TMBPF) and ~20% (CHDM-4-HBA), potentially indicating a different mechanism of antagonism as compared with 4HT and BZA (Fig 2B, grey bars).

Next, we quantified the effect of compound treatment on the E2 induced recruitment of SRC-3, a central ER coregulator, to the PRL array in GFP-ERα:PRL-HeLa (Fig 2E) after 1 hr of treatment. The p160 coactivator SRC-3 is a key coregulator for nuclear receptors and other transcription factors, and has been shown, along with SRC-1, to be significantly recruited to the PRL-array during E2-mediated transcriptional activity [13,15]. As expected, the ER antagonists 4HT and BZA significantly decreased SRC-3 recruitment with %E2 values reduced > 20%. BPA treatment resulted in %E2 values not significantly different from E2 treatment alone. The investigational compounds did not have a significant effect on E2 induced recruitment of SRC-3 to the PRL array, including TMBPF and CHDM-4-HBA. This suggests that while these compounds reduce the ability of ERα to bind DNA in the presence of E2, these compounds do not affect the ability of ERα to recruit SRC-3 once bound to the PRL-array.

We also observed and quantified PRL array targeting of phospho-serine 5 RNA polymerase II (pS5-RNAPII) following 1 hr of compound treatment in the presence of 10 nM E2 (Fig 2E). Phosphorylation at serine 5 identifies RNA polymerase actively transcribing genes, and increases at the PRL array in the presence of E2 [15]. As expected, the ER antagonist 4HT and BZA significantly reduced pS5-RNAPII recruitment by 22% and 38%. Importantly, the only investigational compound to have a significant effect was CHDM-4-HBA, with treatment resulting in the complete loss (100%) of pS5-RNAPII at the PRL array. This was observed despite maintaining SRC-3 recruitment to the PRL array, which is suggestive of a mechanism for an anti-estrogenic activity distinct from that observed with 4HT and BZA.

To investigate potential effects on ER-mediated transcription at the PRL array, we performed mRNA FISH using probes specific to the *dsRED2* reporter protein that is regulated by the PRL promoter elements (S1 Fig). After 1 hour of treatment with 10 nM E2, there was a 4.4-fold increase in de novo *dsRED2* mRNA production (Fig 2F, black bars). Treatment with 5 μM BPA resulted in 60% of the activity observed with E2 treatment whereas none of the investigational compounds activity exceeded 7%. When cells were treated with 10 nM E2 in the presence of 5 μM of the investigational compounds, both CHDM-4-HBA and TMBPF were able to significantly reduce E2-induced de novo mRNA production by 84% and 87%, respectively (Fig 2F, gray bars). This suggests the anti-estrogenic-like effects observed with these compounds on ERα promoter recruitment and/or pS5-RNAPII recruitment leads to reduced ERα mediated transcriptional activity.

Finally, to ensure that results were not due to dramatic changes in GFP-ER expression levels, we quantified GFP-ERα protein level in samples treated with compounds for 24 hr (Fig 2G). Protein levels were measured by quantifying the total GFP intensity per cell in only those cells analyzed for possible visible array formation. Previous studies demonstrated that E2 treatment results in proteasome-dependent degradation of ERα whereas 4HT treatment results in ER stabilization [21]. Consistent with these reports, E2 treatment decreased ERα level by 24% while 4HT and BZA significantly increased protein levels. BPA treatment increased the observed GFP-ERα level by 34%. Of the investigational compounds, both TMBPF-DGE and TMBPF-ER resulted in increased ERα with percent changes of 30% and 20%. The other compounds in this study did not significantly alter the observed GFP-ERα expression level.

### Measure of estrogenic activity using GFP-ERβ:PRL-HeLa cells

To complement the GFP-ERα:PRL-HeLa cell line, we recently generated the GFP-ERβ:PRL-HeLa cell line stably expressing GFP-ERβ. We demonstrated that this cell line responds to E2 in a similar manner as the GFP-ERα:PRL-HeLa cell line and allows for a similar visualization and quantification of multiple aspects of the ERβ transcriptional response [17]. Using the pair of cell lines, we were able to demonstrate differential effects on ERα and ERβ of a panel of BPA analogs [17].

**Fig 3:**
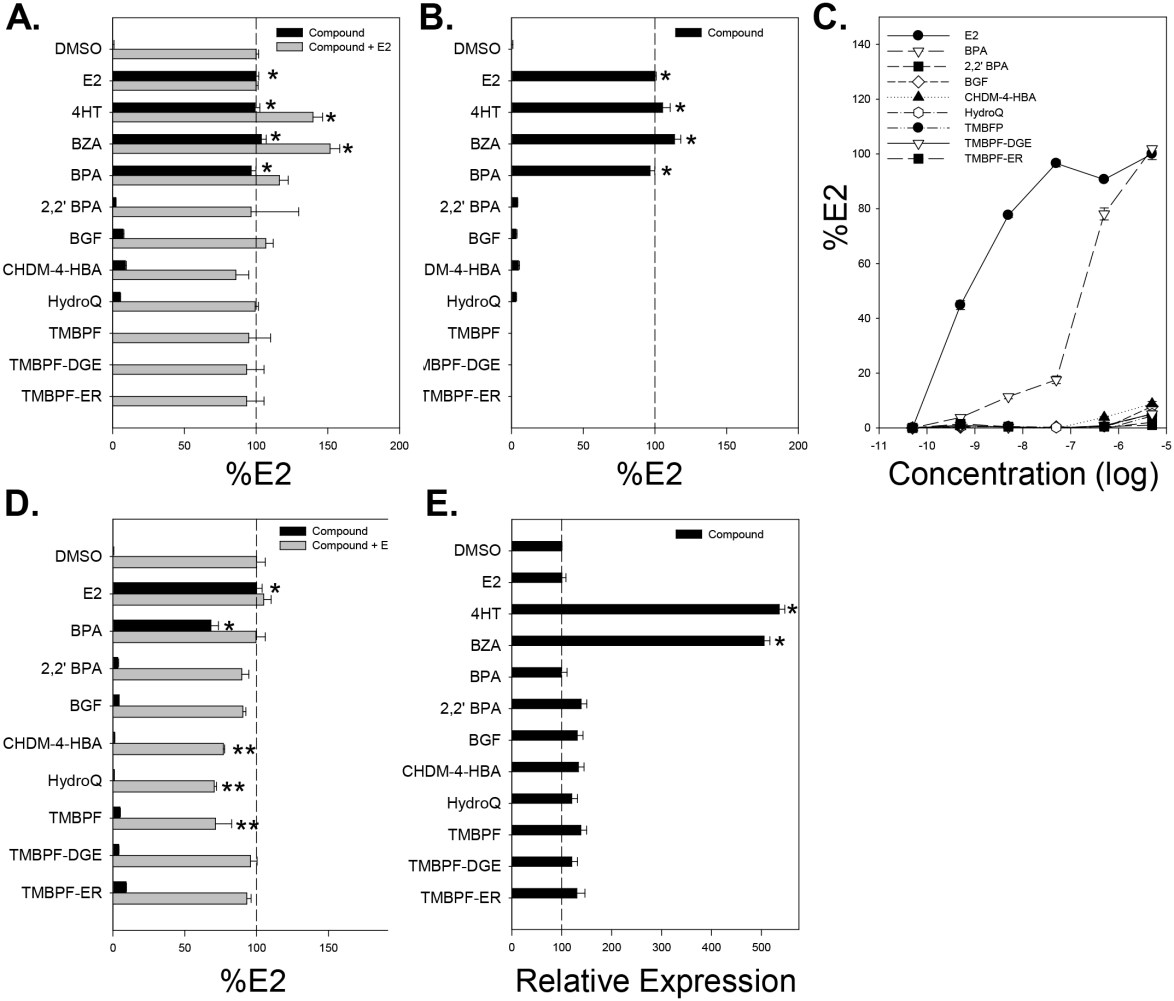
PRL-HeLa analysis of ERβ estrogenic and anti-estrogenic activity of investigational chemical compounds. Percentage of cells with an array in GFP-ERβ:PRL-HeLa cells after 1 hour of treatment with maximal dose of compound alone or with 10 nM E2 after 1 hour (A) or 24 hours (B) of exposure. (C) Dose-response curves of percent of cells with arrays for E2, BPA and the seven investigational compounds (D) Effects on GFP-ERβ expression level after 24 hours of treatment with maximal dose of compound. All data represent mean of a minimum of 4 experiments ± standard deviation. (*) or (**) indicates a response signifcantly (p < 0.05) above or below control treatment.

We treated the GFP-ERβ:PRL-HeLa cells with the compounds for 1 hr (Fig 3A) and determined the %E2 of cells with visible GFP-ERβ recruitment to the PRL array as an indicator of DNA binding. A similar pattern of responses to that observed in the GFP-ERα:PRL-HeLa cell line emerged. Both 10 nM 4HT and 10 nM BZA produced %E2 of 77% and 80%, indicating a strong ability to induce ERβ DNA binding. BPA treatment produced a %E2 score of 76%, similar to that observed with ERα. We observed a very weak but statistically significant ERβ response with 5 μM 2,2’ BPA, CHDM-4-HBA, and TMBPF with %E2 scores ranging between 3% and 9%. The %E2 scores for BGF, HydroQ, TMBPF-DGE, and TMBPF-ER were < 1% and were not significantly different from vehicle. Similar results were observed when treatment time was extended to 24 hr (Fig 3B) or concentrations ranging between 50 pM to 5 μM were tested (Fig 3C).

Similar to ERα, we next determined if compound treatment could alter E2 induced ERβ binding to the PRL array by treating GFP-ERβ:PRL-HeLa with compounds in the presence of 10 nM E2 for 1 hr (Fig 3A, grey bars). As was observed for ERα, 4HT, BZA, and BPA did not inhibit E2 induced ERβ DNA binding. We did not observe the antagonistic effect of TMBPF and CHDM-4-HBA in the ERβ cell line as found in the ERα cell line, suggestive of receptor selectivity. Although CHDM-4-HBA treatment resulted in a %E2 decrease of 11%, this was not significantly different from E2 treatment alone and was dramatically different than the 81% decrease that was observed with ERα.

Although the investigational compounds did not alter GFP-ERβ recruitment to the PRL array, we performed *dsRED2* mRNA FISH to determine if they altered ERβ transcriptional regulation at the PRL promoter. As expected, after 1 hour of treatment both E2 and BPA were able to increase de novo *dsRED2* mRNA production with 10 nM E2 inducing a 3.4-fold increase and BPA treatment reaching 68% of the E2 response (Fig 3D, black bars). When cells were treated with 5 μM of the investigational compounds, no significant increase was observed. In contrast, when compounds were used in combination with 10 nM E2, CHDM-4-HBA, hydroQ, and TMBPF significantly decreased the E2-induced response between 24 to 30% (Fig 3D, gray bars). The anti-estrogenic-like effect of CHDM-4-HBA and TMBPF was markedly-less than what was observed in similar studies using ERα, further suggesting that the effects of these compounds is dependent on the ER isoform present.

Finally, we quantified the GFP-ERβ expression level in GFP-ERβ:PRL-HeLa samples treated with compounds for 24 hr (Fig 3E). In contrast to ERα, E2 and BPA treatment did not have a significant impact on ERβ expression levels. The stabilizing effect of 4HT and BZA was consistent across receptor isotypes, with treatment resulting in a percent change of 436% and 405%, respectively. None of the investigational compounds altered ERβ expression levels when cells were treated up to 5 μM.

### Measure of estrogenic activity using an integrated ERE-luciferase reporter

Although the image based approach using the prolactin array cell line allows for an intensive and mechanistic interrogation of estrogenic activity at the level of individual cells, the HeLa cell line does not endogenously express ER and therefore is not inherently responsive to estrogenic compounds. To overcome this limitation using a routine population-based approach, we used an estrogen responsive MCF-7 line containing a genomic integration of an ERE-driven firefly luciferase reporter to characterize the compounds of interest.

**Fig 4:**
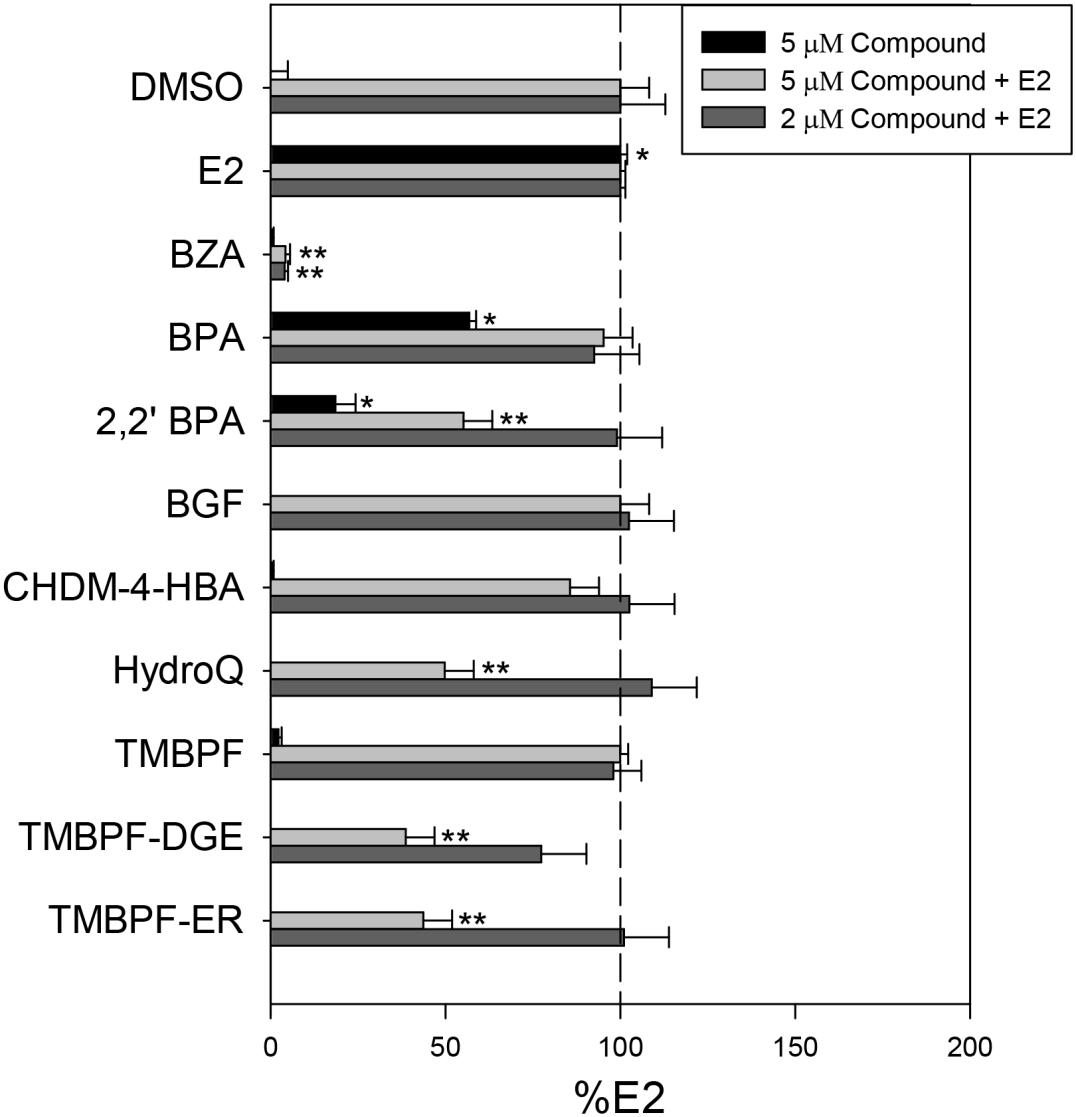
Chemical compound effect on an integrated ERE-driven luciferase reporter in the ER responsive MCF-7 cell line. MCF-7-MAR-ERE-Luc were treated with indicated compounds at 5 μM, 5 μM + 10 nM E2, or 2 μM + 10 nM E2 for 24 hours before total luciferase activity determined. The %E2 calculated using control wells treated with E2 (10 nM) or DMSO. Dashed line indicates 10 nM E2 control response. All data represent mean of 3 experiments ± standard deviation. (*) or (**) indicates a response significantly (p < 0.05) above or below control treatment.

Treatment of the MCF-7 ERE-MAR cell line with 10 nM E2 for 24 hours resulted in a ~19-fold induction of luciferase activity (data not shown) whereas treatment with 2 μM BZA resulted in a %E2 luciferase activity score < 1% (Fig 4). BPA treatment resulted in a significant %E2 luciferase score of 57%. When tested at 5 μM, a majority of the investigational compounds did not result in a %E2 luciferase score significantly different from vehicle (Fig 4), which validates the results from the PRL array cell line. However, 2,2’ BPA treatment resulted in a %E2 of 19%, which suggest the presence of weak estrogenic activity in this assay.

When MCF-7 ERE-MAR cells were treated with 10 nM E2 in the presence of the compounds to determine potential antagonistic activity, several were found to have an effect (Fig 4). As expected, the ER antagonist BZA decreased E2 induced luciferase by > 95%. Treatment with 5 μM BPA did not significantly reduce the E2 response, as expected. With the investigational compounds, there was an observable decrease in cell number following treatment when directly examined, indicating a possible toxic effect at this dose in MCF-7 cells that was not observed in the HeLa lines. The compounds TMBPF-DGE, TMBPF-ER, HydroQ, and 2,2’ BPA caused a significant inhibition with 41–61% reduction in luciferase activity. Further, TMBPF and CHDM-4-HBA also resulted in an observable decrease; however, these results were not statistically different from E2 alone. When concentrations of the investigational compounds were reduced to 2 μM, no evidence of cell loss was observed and there was no detectable anti-estrogenic-like activity; collectively, these data suggest that the previous results were likely due to non-specific toxicity effects.

### Measure of androgenic activity using an integrated probasin-mCherry-NLS reporter

Computational structural analysis, animal studies, and in vitro assays have all generated evidence that BPA and BPA analogs are capable of altering androgenic signaling through direct interactions with the androgen receptor (AR) [22–25]. To assay potential androgenic- or anti-androgenic-like activity of the investigational compounds in this study, we used the androgen responsive LNCaP prostate cancer cell line with a stable integration of a probasin promoter element (2PB-mChrry-NLS:LNCAP) that contains multiple androgen response elements (AREs), and regulates expression of a mCherry-NLS-mCherry fluorescent protein. The parental LNCaP cell line contains a T877A mutation in the ligand binding domain of AR which is known to increase the receptor responsiveness to estrogens and the anti-androgen hydroxyflutamide.

**Fig 5:**
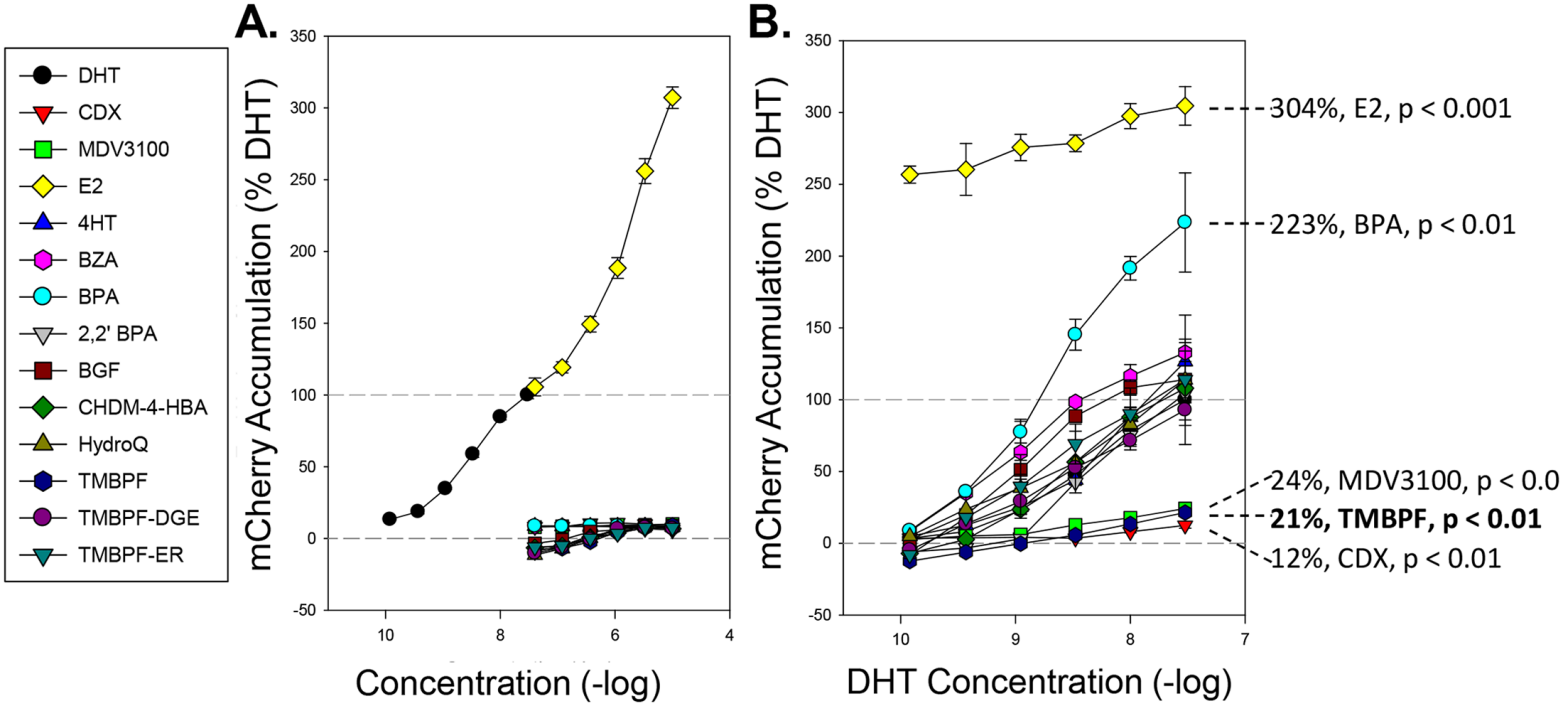
Chemical compound effect on an integrated ARE-containing probasin-mCherry-NLS reporter in the AR responsive LNCaP cell line. The nuclear accumulation of mCherry fluorescent protein in 2PB-mCherry-NLS:LNCaP cells treated with indicated compounds either alone at concentrations from 50 pM to 5 μM (A) or at 5μM with DHT at ranging from 0.1 nM to 30 nM (B) for 48 hours. Compounds that induced either a significant androgen-like or anti-androgen-like response in the presence of DHT are indicated. All data represent mean of 3 experiments ± standard deviation.

As expected, DHT treatment was able to induce a dose dependent increase in the nuclear accumulation of the reporter protein, with a 4-fold increase observed after 48 h treatment with 30 nM DHT (Fig 5A). A strong response was also observed with E2 treatment due to the T877A mutation. We were unable to observe any androgenic-like activity from BPA or the investigational compounds after 48 hours of treatment. Next, cells were treated with increasing concentrations of DHT in the presence of 5 μM of reference or investigational compounds for 48 hours (Fig 5B). Again, we observed a strong androgenic-like effect of E2 in this cell line. Further, we were able to observe a weaker, but significant androgenic-like effect of BPA treatment. The known anti-androgens casodex (CDX, bicalutamide) and MDV3100 (enzalutamide) were able to decrease the DHT induced response by 88 and 76%. Importantly, the anti-estrogens 4HT and BZA did not significantly alter the DHT response; however, the investigational compound TMBPF resulted in a significant 79% decrease in the DHT induced response indicating a strong anti-androgenic-like response.

### Clustering analysis of estrogen-like activity across all assays

**Fig 6:**
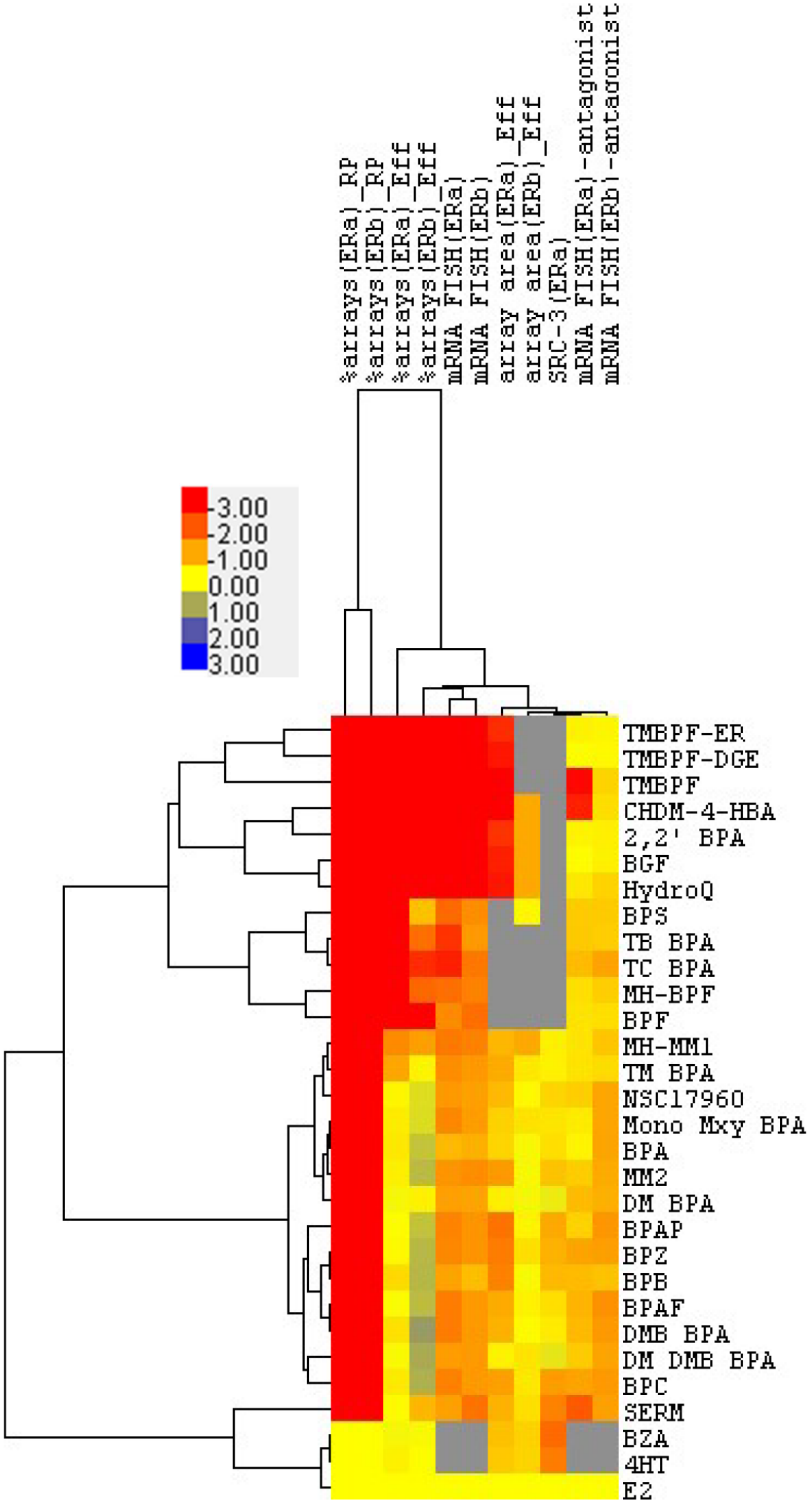
Clustering analysis of investigational chemical compounds and previously studied bisphenol analogs activity across multiple *in vitro* assays. Data in each assay row were range normalized before clustering analysis. Clustering was performed using Euclidean distance for both the compounds and the assays.

To gain a more comprehensive view of these disparate data sets, we compiled the above results and included previously published[17] data that were used to characterize a panel of BPA analogs (*i*.*e*., “BPX” compounds). We standardized all data to E2 treated controls. Because the antagonistic effects on E2 induced DNA binding, ER expression, and pS5-RNAPII recruitment were not examined in the published BPX work, these metrics were excluded from the cluster analysis. The resulting data set was range normalized and clustered using Euclidean distance (Fig 6). Analysis of the clustering reveals that E2 and mixed agonist/antagonist treatments (4HT, BZA) result in a distinct response pattern and suggest that neither the BPX compounds nor the investigational compounds in this study exhibit E2- or SERM-like activity. The second major cluster includes BPA and multiple BPA structural analogs, compounds that retain a significant degree of estrogenic activity. The investigational compounds 2,2’ BPA, BGF, CHDM-4-HBA, TMBPF, TMBPF-DGE, and TMBPF-DGE cluster into a branch distant from that containing BPA. The investigational compounds are further subdivided into smaller branches based upon specific functional differences, such as minor differences in induced ERβ DNA binding and inhibition of E2 induced transcription.

## Discussion

In response to concerns regarding BPA, there is an emphasis on manufacturing products that no longer use BPA. In some cases, BPA has been replaced with analogs such as BPS and BPF; consequently, human exposure to these analogs is now approaching the same levels as BPA [8]. It is becoming clear that BPA analogs can have similar effects as BPA in cell-based *in vitro* assays and *in vivo* animal models [8,17,24,26,27]. These results emphasize the need for rigorous studies to understand potential endocrine effects of compounds proposed as BPA alternatives before widespread adoption. To this end, we completed a set of cell-based studies that included quantitative and mechanistic metrics of estrogenic and androgenic activity on a set of compounds previously identified using a yeast-based *in vitro* screening approach as having minimal estrogen-like properties.

When utilizing the PRL-HeLa and MCF-7 cell models to characterize a set of reference compounds and seven investigational compounds (2’,2 BPA, BGF, CHDM-4-HBA, HydroQ, TMBPF, TMBPF-DGE, and TMBPF-ER), the estrogenic effects of BPA were clear and consistent with previous work. In PRL-HeLa cells expressing either GFP-ERα or GFP-ERβ, BPA exposure resulted in an extent of visible array formation comparable with E2 treatment. This indicates that at a sufficient concentration, BPA exposure results in the efficient binding of ER (α or β) to the estrogen response sequences located in the prolactin promoter, a crucial step in estrogen regulated gene expression. This result was confirmed by the significant induction of de novo mRNA production at the PRL array and ERE-driven reporter gene activity in the estrogen responsive MCF-7 breast cancer cell line.

In contrast, none of the seven investigational compounds were able to induce significant promoter binding in PRL-HeLa cells expressing either GFP-ERα or GFP-ERβ when tested up to micromolar concentrations at both short (1 hr) and long (24 hr) time points. Based upon hierarchical clustering using the assays presented, these 7 compounds populate a cluster distinct from BPA and 18 other previously- studied BPX compounds and are marked by a relative lack of estrogenic activity (Fig 5). Within in this cluster, 2,2’ BPA is unique in that it is characterized by weak estrogenic activity in the MCF-7 cell line with little to no activity in the PRL-HeLa cell lines. This indicates that the altered position of the hydroxyl groups results in a receptor-ligand interaction that retains residual activity on the basic ERE element contained in the MCF-7 reporter cell line, but is insufficient to drive estrogenic activity in the context of the prolactin promoter elements used in the PRL-HeLa cell lines.

Unique within this cluster are the responses observed with CHDM-4-HBA and TMBPF. These are the first compounds we have observed that demonstrated any ability to alter the promoter binding induced by 10 nM E2, reducing ERα recruitment to the PRL array by ~80% and ~40% and significantly reducing de novo mRNA production. This is in contrast to well-studied compounds with anti-estrogenic activity such as 4HT and BZA, which antagonize the E2 transcriptional response by the recruitment of corepressor complexes that inhibit transcription, but do not alter ERα recruitment to the PRL array [15,17]. The effect was greatest in the GFP-ERα:PRL-HeLa cell line and not observed or significantly reduced with the GFP-ERβ:PRL-HeLa and the MCF-7 cell lines. In addition, only TMBPF demonstrated an effect on the androgen induced transcriptional activity of the promiscuous AR receptor expressed in the LNCaP prostate cancer cell line. These results suggest that these effects on transcriptional activity are selective based upon receptor, receptor subtype preference, and/or cell background. When promoter binding was observed, neither compound altered the recruitment of the ER coregulator SRC-3 and only CHDM-4-HBA decreased levels of activated RNA Pol II at the integrated PRL array. One potential mechanism by which these compounds may be acting is through the activation of other type I nuclear receptors, as others have observed that activation of the glucocorticoid receptor GR can displace ER from DNA binding sites [28]. The PRL-HeLa cell line does express endogenous GR; however, we have shown that direct activation of endogenous GR using dexamethasone increases, not decreases, ERα recruitment to the PRL array which suggests this mechanism is not involved in the responses observed with CHDM-4-HBA and TMBPF [19]. Therefore, the mechanisms behind these observations remain unknown, and importantly, it has been subsequently shown that TMBPF and its related compounds do not show biological effects in either E-SCREEN or mouse uterotorphic in vivo assays [29].

The remaining four investigational compounds, including two TMBPF derivatives (BGF, TMBPF-DGE, TMBPF-ER, and HydroQ), demonstrated little activity in the *in vitro* assays for estrogenic, anti-estrogenic, androgenic, and anti-androgenic activity. Although a significant decrease in E2 induced reporter gene activity in the MCF-7 cell line was observed with 5 μM TMBPF-DGE, TMBPF-ER, and HydroQ, significant cell toxicity was also observed. When the concentration was reduced from 5 μM to 2 μM, cell toxicity was absent, and there was no observable effect on E2 induced reporter gene activity. This suggests that the results observed at the higher tested concentration were due to non-specific cell toxicity and not a specific anti-estrogenic-like activity associated with the compounds. Although CHDM-4-HBA appeared to demonstrate anti-estrogenic-like activity in the PRL-HeLa assays, this result was not supported in the ERE-luciferase assays performed using the MCF-7 cell line that endogenously expresses ERα.

Considering these findings, it appears several of these investigational compounds, especially HydroQ, BGF and the TMBPF derivatives TMBPF-DGE and TMBPF-ER, demonstrate little of the estrogen-like activity shown by BPA and various BPA analogs. The TMBPF findings are consistent with a recent publication in which this compound was found to not have estrogenic-like properties in an *in vivo* uterotrophic assay [29]. HydroQ, a compound with evidence of weak anti-estrogen-like activity in the GFP-ERβ:PRL-HeLa cell line, is the only investigational compound that has been included in the EPA ToxCast program where it was found to have weak estrogenic activity at concentrations (EC_50_ > 30 μM) exceeding those tested in this study [30]. There is little to no existing data describing the estrogenic or anti-estrogenic activity of BGF, TMBFP-DGE and TMBPF-ER, compounds that may represent useful alternatives to BPA. For example, the lack of activity by BGF is exciting as this is a “green” compound readily synthesized from plant material found in by-products from the paper industry [31]. Based upon the lack of activities demonstrated here and minimal pre-existing data, these compounds are worthwhile candidates for further investigation using an expanded panel of *in vitro* assays such as those sanctioned by the EPA and used in current models predicting estrogenic activity [12], and/or using *in vivo* models to further our understanding as suitable BPA alternatives.

## Supporting information

S1 Data Source and raw data for summary figures. Excel worksheet containing oringal and normalized data used to generate summary figures.
(XLSX)

S1 Fig Prolactin integrated promoter array model system. (A) Schema showing the essential elements of the reporter constructions. Transcription start site, proximal promoter and enhancer sequence are shown. (B) Multi-copy integration in a HeLa cell line stably expressing GFP-tagged ER allows visualization of estrogen induced binding as a bright intra-nuclear spot of varying size/shape/texture linked to transcriptional activity (Bolt et al, 2014; Stossi et al, 2014). Red lines indicate array mask (1) and cell mask (2) generated by image analysis algorithms and allow quantification of features listed. Examples of samples treated with either 10 nM estradiol (C) or non-estrogenic 5 μM 2,2’ BPA (D).
(TIF)

## References

[1] Olea N, Pulgar R, Pérez P, Olea-Serrano F, Rivas A, Novillo-Fertrell A, et al Estrogenicity of resin-based composites and sealants used in dentistry. Environ Health Perspect. 1996;104: 298–305. 8919768 10.1289/ehp.96104298 PMC1469315

[2] Brotons JA, Olea-Serrano MF, Villalobos M, Pedraza V, Olea N. Xenoestrogens released from lacquer coatings in food cans. Environ Health Perspect. 1995;103: 608–12. 7556016 10.1289/ehp.95103608 PMC1519121

[3] Welshons W V, Thayer KA, Judy BM, Taylor JA, Curran EM, vom Saal FS. Large effects from small exposures. I. Mechanisms for endocrine-disrupting chemicals with estrogenic activity. Environ Health Perspect. 2003;111: 994–1006. 12826473 10.1289/ehp.5494 PMC1241550

[4] Calafat AM, Ye X, Wong L-Y, Reidy JA, Needham LL. Exposure of the U.S. population to bisphenol A and 4-tertiary-octylphenol: 2003–2004. Environ Health Perspect. 2008;116: 39–44. doi: 10.1289/ehp.10753 18197297 10.1289/ehp.10753 PMC2199288

[5] Weiss B. The intersection of neurotoxicology and endocrine disruption. Neurotoxicology. 2012;33: 1410–9. doi: 10.1016/j.neuro.2012.05.014 22659293 10.1016/j.neuro.2012.05.014 PMC3458140

[6] Rochester JR. Bisphenol A and human health: a review of the literature. Reprod Toxicol. 2013;42: 132–55. doi: 10.1016/j.reprotox.2013.08.008 23994667 10.1016/j.reprotox.2013.08.008

[7] Braun J., Lanphear BP, Calafat AM, Deria S, Khoury J, Howe CJ, et al Early-Life Bisphenol A Exposure and Child Body Mass Index: A Prospective Cohort Study. Environ Health Perspect. 2014;122: 1239–1245. doi: 10.1289/ehp.1408258 25073184 10.1289/ehp.1408258 PMC4216170

[8] Rochester J, Bolden A. Bisphenol S and F: A systematic Review and Comparison of the Hormanal Activity of Bisphenol A Substitutes. Environ Health Perspect. 2015;123: 643–50. doi: 10.1289/ehp.1408989 25775505 10.1289/ehp.1408989 PMC4492270

[9] Odian GG. Principles of polymerization.

[10] Robertson GL. Food packaging: principles and practice. Taylor & Francis/CRC Press; 2006.

[11] Rogers JM, Denison MS. Recombinant cell bioassays for endocrine disruptors: development of a stably transfected human ovarian cell line for the detection of estrogenic and anti-estrogenic chemicals. In Vitr Mol Toxicol. 2000;13: 67–82. 10900408

[12] Judson RS, Magpantay FM, Chickarmane V, Haskell C, Tania N, Taylor J, et al Integrated Model of Chemical Perturbations of a Biological Pathway Using 18 In Vitro High Throughput Screening Assays for the Estrogen Receptor. Toxicol Sci. 2015;148: kfv168. doi: 10.1093/toxsci/kfv168 26272952 10.1093/toxsci/kfv168 PMC4635633

[13] Sharp ZD, Mancini MG, Hinojos C a., Dai F, Berno V, Szafran AT, et al Estrogen-receptor- exchange and chromatin dynamics are ligand- and domain-dependent. J Cell Sci. 2006;119: 4365–4365. doi: 10.1242/jcs.03264 10.1242/jcs.03161 16968748

[14] Berno V, Amazit L, Hinojos C, Zhong J, Mancini MG, Sharp ZD, et al Activation of estrogen receptor-alpha by E2 or EGF induces temporally distinct patterns of large-scale chromatin modification and mRNA transcription. PLoS One. 2008;3: e2286 doi: 10.1371/journal.pone.0002286 18509470 10.1371/journal.pone.0002286 PMC2386239

[15] Ashcroft FJ, Newberg JY, Jones ED, Mikic I, Mancini M a. High content imaging-based assay to classify estrogen receptor-α ligands based on defined mechanistic outcomes. Gene. Elsevier B.V.; 2011;477: 42–52. doi: 10.1016/j.gene.2011.01.009 21256200 10.1016/j.gene.2011.01.009 PMC3086628

[16] Szafran AT, Mancini MA. The myImageAnalysis project: a web-based application for high-content screening. Assay Drug Dev Technol. 2014;12: 87–99. doi: 10.1089/adt.2013.532 24547743 10.1089/adt.2013.532 PMC3934667

[17] Stossi F, Bolt MJ, Ashcroft FJ, Lamerdin JE, Melnick JS, Powell RT, et al Defining estrogenic mechanisms of bisphenol A analogs through high throughput microscopy-based contextual assays. Chem Biol. Elsevier Ltd; 2014;21: 743–753. doi: 10.1016/j.chembiol.2014.03.013 24856822 10.1016/j.chembiol.2014.03.013 PMC4301571

[18] Stossi F, Dandekar RD, Bolt MJ, Newberg JY, Mancini MG, Kaushik AK, et al High throughput microscopy identifies bisphenol AP, a bisphenol A analog, as a novel AR down-regulator. Oncotarget. 2016; doi: 10.18632/oncotarget.7655 26918604 10.18632/oncotarget.7655 PMC4941363

[19] Bolt MJ, Stossi F, Newberg JY, Orjalo A, Johansson HE, Mancini M a. Coactivators enable glucocorticoid receptor recruitment to fine-tune estrogen receptor transcriptional responses. Nucleic Acids Res. 2013;41: 4036–48. doi: 10.1093/nar/gkt100 23444138 10.1093/nar/gkt100 PMC3627592

[20] Jiang S, Meyer R, Kang K, Osborne C, Wong J, Oesterreich S. Scaffold attachment factor SAFB1 suppresses estrogen receptor alpha-mediated transcription in part via interaction with nuclear receptor corepressor. Mol Endocrinol. 2006;20: 311–320. doi: 10.1210/me.2005-0100 16195251 10.1210/me.2005-0100

[21] Stenoien DL, Patel K, Mancini MG, Dutertre M, Smith CL, O’Malley BW, et al FRAP reveals that mobility of oestrogen receptor-alpha is ligand- and proteasome-dependent. Nat Cell Biol. 2001;3: 15–23. doi: 10.1038/35050515 11146621 10.1038/35050515

[22] Rehan M, Ahmad E, Sheikh IA, Abuzenadah AM, Damanhouri GA, Bajouh OS, et al Androgen and Progesterone Receptors Are Targets for Bisphenol A (BPA), 4-Methyl-2,4-bis-(P-Hydroxyphenyl)Pent-1-Ene—A Potent Metabolite of BPA, and 4-Tert-Octylphenol: A Computational Insight. PLoS One. 2015;10: e0138438 doi: 10.1371/journal.pone.0138438 26379041 10.1371/journal.pone.0138438 PMC4574962

[23] Wu J, Huang D, Su X, Yan H, Sun Z. Oral administration of low-dose bisphenol A promotes proliferation of ventral prostate and upregulates prostaglandin D2 synthase expression in adult rats. Toxicol Ind Health. 2015; doi: 10.1177/0748233715590758 26088557 10.1177/0748233715590758

[24] Rosenmai AK, Dybdahl M, Pedersen M, Alice van Vugt-Lussenburg BM, Wedebye EB, Taxvig C, et al Are Structural Analogues to Bisphenol A Safe Alternatives? Toxicol Sci. 2014;139: 35–47. doi: 10.1093/toxsci/kfu030 24563381 10.1093/toxsci/kfu030

[25] Colleta SJ, Antoniassi JQ, Zanatelli M, Santos FCA, Góes RM, Vilamaior PSL, et al Acute exposure to bisphenol A and cadmium causes changes in the morphology of gerbil ventral prostates and promotes alterations in androgen-dependent proliferation and cell death. Environ Toxicol. 2015; doi: 10.1002/tox.22211 26537420 10.1002/tox.22211

[26] Kitamura S, Suzuki T, Sanoh S, Kohta R, Jinno N, Sugihara K, et al Comparative study of the endocrine-disrupting activity of bisphenol A and 19 related compounds. Toxicol Sci. 2005;84: 249–59. doi: 10.1093/toxsci/kfi074 15635150 10.1093/toxsci/kfi074

[27] Rivas A, Lacroix M, Olea-Serrano F, Laíos I, Leclercq G, Olea N. Estrogenic effect of a series of bisphenol analogues on gene and protein expression in MCF-7 breast cancer cells. J Steroid Biochem Mol Biol. 2002;82: 45–53. 12429138 10.1016/s0960-0760(02)00146-2

[28] Karmakar S, Jin Y, Nagaich AK. Interaction of glucocorticoid receptor (GR) with estrogen receptor (ER) α and activator protein 1 (AP1) in dexamethasone-mediated interference of ERα activity. J Biol Chem. 2013;288: 24020–34. doi: 10.1074/jbc.M113.473819 23814048 10.1074/jbc.M113.473819 PMC3745347

[29] Soto AM, Schaeberle C, Maier MS, Sonnenschein C, Maffini M V. Evidence of Absence: Estrogenicity Assessment of a New Food-Contact Coating and the Bisphenol Used in Its Synthesis. Environ Sci Technol. American Chemical Society; 2017;51: 1718–1726. doi: 10.1021/acs.est.6b04704 28098991 10.1021/acs.est.6b04704

[30] Dix DJ, Houck KA, Martin MT, Richard AM, Setzer RW, Kavlock RJ. The ToxCast program for prioritizing toxicity testing of environmental chemicals. Toxicol Sci. 2007;95: 5–12. doi: 10.1093/toxsci/kfl103 16963515 10.1093/toxsci/kfl103

[31] Stoye E. BPA substitute made from paper industry leftovers. In: Chemistry World [Internet]. 2014. http://www.rsc.org/chemistryworld/2014/03/green-safe-bpa-substitute-bgf-lignin-paper

