# Supplementary figures and images for: Characterizing properties of non-estrogenic substituted bisphenol analogs using high throughput microscopy and image analysis
Source: PLoS One. 2017 Jul 13;12(7):e0180141. doi: 10.1371/journal.pone.0180141 (PMC5509144; doi:10.1371/journal.pone.0180141)

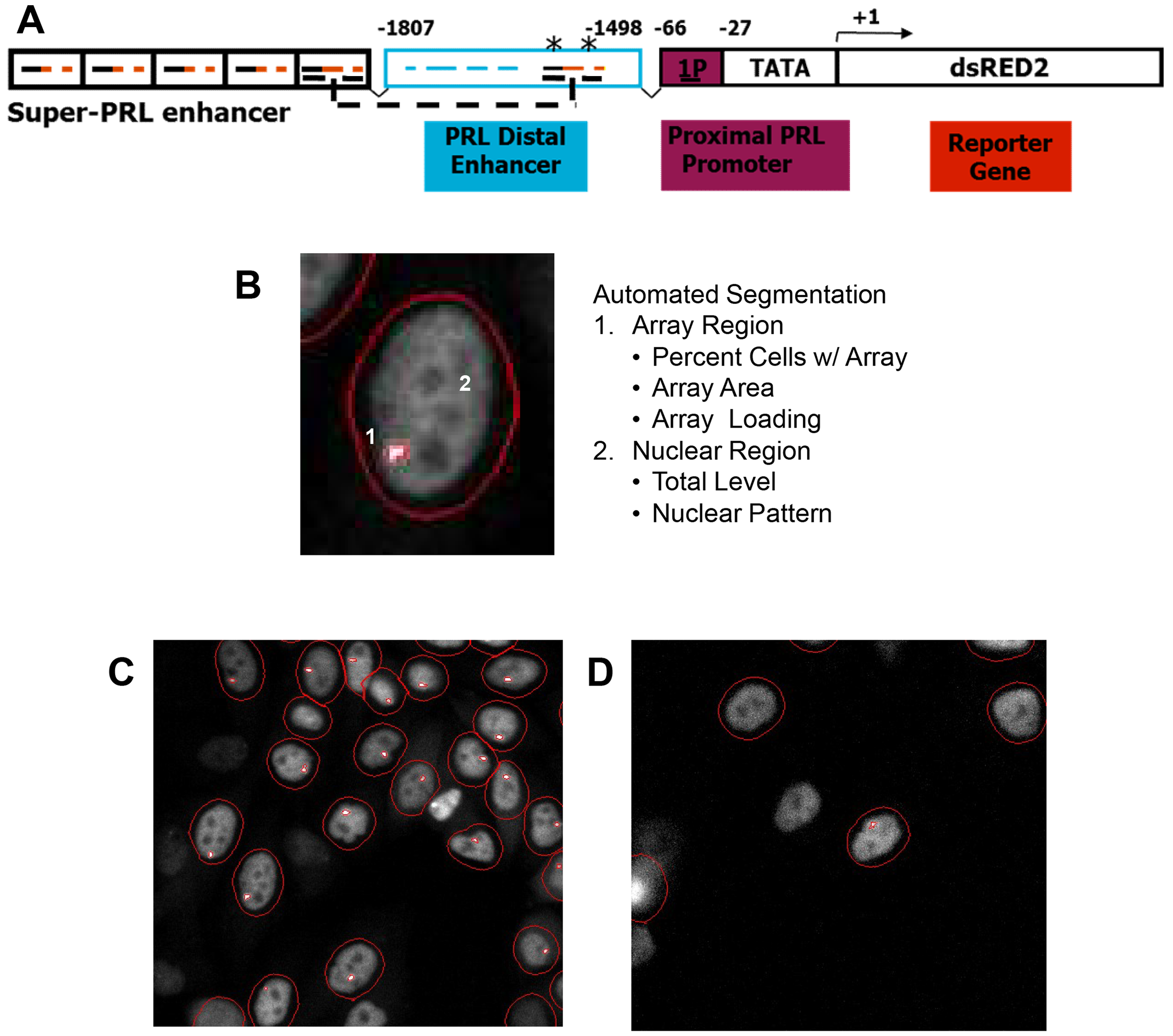

Supplement: S1 Fig — (A) Schema showing the essential elements of the reporter constructions. Transcription start site, proximal promoter and enhancer sequence are shown. (B) Multi-copy integration in a HeLa cell line stably expressing GFP-tagged ER allows visualization of estrogen induced binding as a bright intra-nuclear spot of varying size/shape/texture linked to transcriptional activity (Bolt et al, 2014; Stossi et al, 2014). Red lines indicate array mask (1) and cell mask (2) generated by image analysis algorithms and allow quantification of features listed. Examples of samples treated with either 10 nM estradiol (C) or non-estrogenic 5 μM 2,2’ BPA (D). (TIF) [file pone.0180141.s002.tif]
